# Low impact of *Zostera marina* meadows on sediment and water microbiota under brackish conditions

**DOI:** 10.1186/s40793-024-00662-6

**Published:** 2025-01-11

**Authors:** Daniel P. R. Herlemann, Luis F. Delgado, David J. Riedinger, Víctor Fernández-Juárez, Anders F. Andersson, Christian Pansch, Lasse Riemann, Mia M. Bengtsson, Greta Gyraitė, Marija Kataržytė, Veljo Kisand, Sandra Kube, Georg Martin, Kasia Piwosz, Marcin Rakowski, Matthias Labrenz

**Affiliations:** 1 Biological Oceanography, Leibniz Institute for Baltic Sea Research Warnemünde (IOW), 18119 Rostock, Germany; 2 Science for Life Laboratory, School of Biotechnology, Division of Gene Technology, KTH Royal Institute of Technology, Solna, 171 21 Sweden; 3 Department of Biology, University of Copenhagen, Helsingør, 3000 Denmark; 4 Center for Limnology, Estonian University of Life Sciences, Tartu, 51006 Estonia; 5 Faculty of Science and Engineering, Environmental and Marine Biology, Åbo Akademi University, Turku/Åbo, 20500 Finland; 6 Institute of Microbiology, University of Greifswald, 17489 Greifswald, Germany; 7 Marine Research Institute, Klaipėda University, Klaipėda, 92294 Lithuania; 8 Institute of Biosciences, Life Sciences Center, Vilnius University, Vilnius, 10257 Lithuania; 9 Estonian Marine Institute, University of Tartu, Tallinn, 12618 Estonia; 10 National Marine Fisheries Research Institute, Gdynia, 81-332 Poland

**Keywords:** Coastal zone, Salinity, Horohalinicum, Baltic Sea, Bacterial community, Microeukaryotic community, Seagrass, Littoral, Eelgrass

## Abstract

**Background:**

*Zostera marina* is an important ecosystem engineer influencing shallow water environments and possibly shaping the microbiota in surrounding sediments and water. *Z. marina* is typically found in marine systems, but it can also proliferate under brackish conditions. Changes in salinity generally have a strong impact on the biota, especially at the salty divide between salinity 6 and 9. To better understand the impact of the salty divide on the interaction between *Z. marina* and the surrounding sediment and water microbiota, we investigated the effects of *Z. marina* meadows on the surrounding microbiota across a salinity range of 6–15 in the Baltic Sea during the summer using 16S and 18S rRNA gene amplicon sequencing.

**Results:**

Salinity was the most important factor for structuring the microbiota within both water and sediment. The presence of *Z. marina* affected the composition of the bacterial and eukaryotic community and bacterial alpha diversity in the sediment. However, this effect was confined to alpha-mesohaline conditions (salinity 9–15). The impact of *Z. marina* below salinity 9 on water and sediment microbiota was insignificant.

**Conclusions:**

Increasing salinity was associated with a longer leaf length of *Z. marina*, causing an increased canopy height, which affects the sediment microbiota through reduced water velocity. Hence, we propose that the canopy effect may be the major predictor explaining *Z. marina*’s interactions with the surrounding microbiota at salinity 9–15. These findings emphasize the importance of the physical effects of *Z. marina* meadow ecosystem services and have important implications for *Z. marina* management under brackish conditions in a changing climate.

**Supplementary Information:**

The online version contains supplementary material available at 10.1186/s40793-024-00662-6.

## Introduction

In shallow, sheltered coastal regions, *Zostera marina* (also called eelgrass) builds large meadows that provide ecosystem services, including sediment stabilization, food provision, shelter, and nutrient recycling. It maintains intimate ecological interactions with microbial consortia that live in association with plants and within the surrounding seawater and sediments [1, 2]. Despite their ecological importance, *Z. marina* meadows decline globally by 2–5% annually, primarily due to human pressures, including climate change [3, 4]. *Z. marina* is widespread along coastlines throughout the Northern Hemisphere and has been suggested as a model system for aquatic plants [5]. Because the leaves and roots are constantly submerged, microorganisms attached to *Z. marina* interact strongly with the surrounding water and sediment bacterial communities [2, 6]. For example, the release of organic molecules (e.g., amino acids and sugars) and gases (CO_2_, N_2_) by aquatic macrophytes is associated with the presence of specific sulfate-reducing and nitrogen-fixing bacterial communities [7–10]. However, interactions with the surrounding environment extend beyond directly attached bacteria and may also be recognized within the *Z. marina* meadow, e.g., by the bacterial community in the sediment surrounding the roots [11–13] as well as the edges of *Z. marina* meadow patches [5].

In contrast to the effects on sediment, the influence of *Z. marina* on bacteria in the water columns of its meadows is still debated and seems to depend on environmental conditions [14–18]. For instance, based on a reduction in colony-forming bacterial units in the intertidal regions of tropical islands, Lamb et al. [19] suggested that *Z. marina* acts as a natural water filtration system for allochthonous pathogenic bacteria. Similarly, other studies have found lower levels of potentially pathogenic bacteria in *Z. marina* meadows than in areas without *Z. marina* [20–23]. Tasdemir et al. [24] and Millan et al. [25] reported the presence of potentially antibiotic-producing bacteria in *Z. marina* leaves, suggesting an influence on the surrounding seawater. However, yet other studies did not provide conclusive evidence of pathogen reduction by *Z. marina* in coastal waters [14, 15, 26]. Hence, the interaction between *Z. marina* and the surrounding bacterial community is not fully understood [14–16, 18].

In this study, we characterized the water and sediment microbiota inside, on the edge of, and outside of *Z. marina* meadows at different salinity levels at Baltic Sea coastal sites. *Z. marina* meadows are present within a salinity range of 5–35. Within this salinity range, especially at the salty divide at salinities 6–9, the sediment and water microbiota change from marine-related to freshwater-related organisms [27–31]. The salinity-driven shift in the biota at the salty divide [29, 32, 33] is a fundamental concept in ecology that has effects on species richness and community composition in different biological groups [34]. Few studies have investigated the effects of the host and its surrounding microbiota among the salty divide [35, 36]. Previous studies investigating the impact of salinity on bacterial communities associated with macrophytes suggested that host and habitat were the most important factors in structuring their composition [37, 38], with the strongest changes occurring in the salty divide [39, 40]. We hypothesized that (1) the direct effect of *Z. marina* on the sediment and water microbiota differs depending on the salinity and that (2) the effect of *Z. marina* on the meadow water and sediment microbiota decreases with increasing distance from the *Z. marina* meadow. To address these hypotheses, we investigated *Z. marina* meadows in the Baltic Sea, where a stable salinity gradient with a characteristic sediment and water bacterial community exists [29, 30] and where *Z. marina* is highly abundant along the salinity gradient [41].

## Materials and methods

**Fig. 1:**
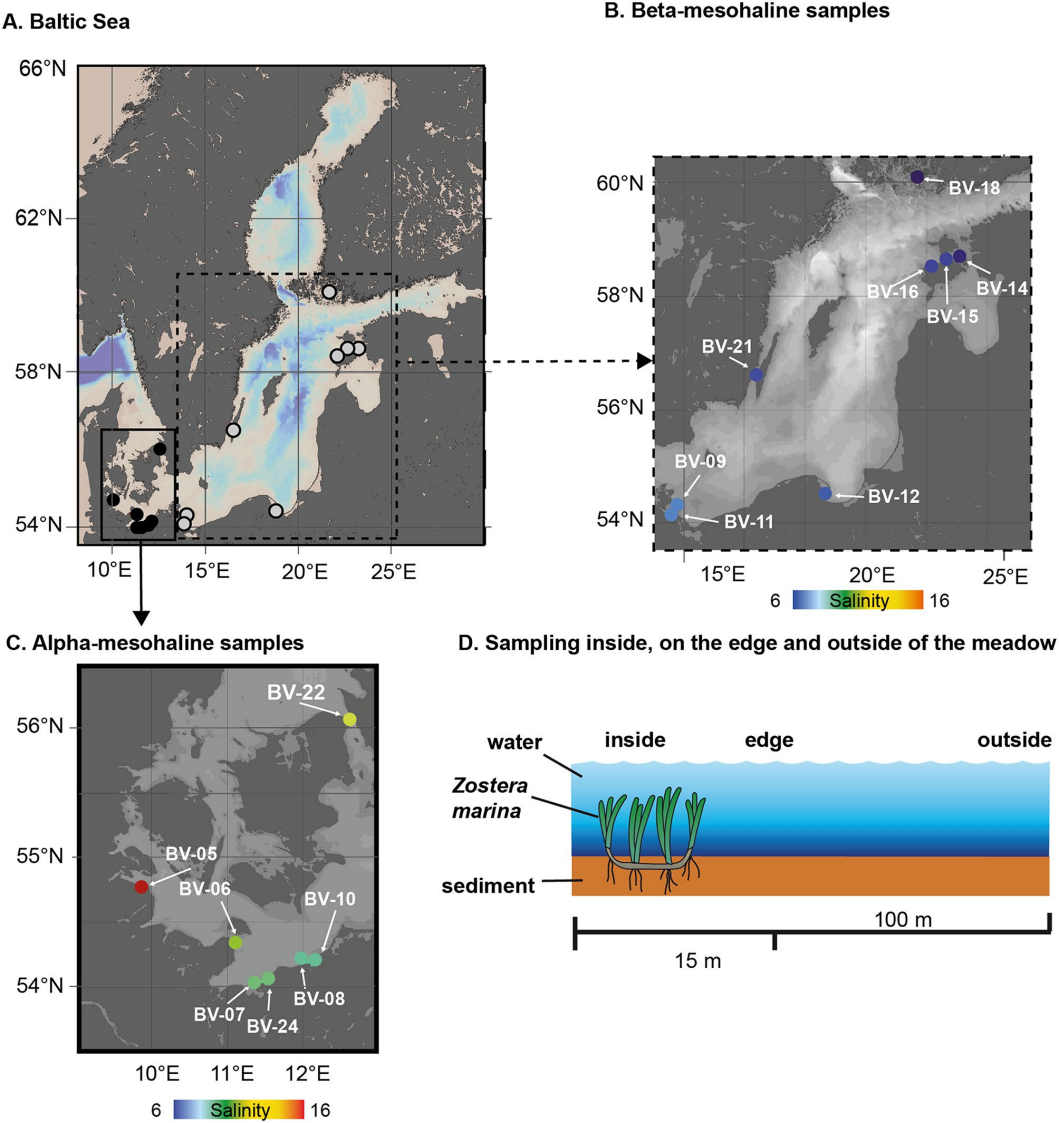
Sampling location (**A**) alpha-mesohaline stations colored by salinity; (**B**) overview of the Baltic Sea sites where the subgraphs are marked (**C**) beta-mesohaline stations colored by salinity. (**D**) Sampling scheme for each station. Samples were collected inside the *Zostera marina* field, on the edge (15 m), and outside (100 m) of the *Z. marina* field

Samples were collected as described by Riedinger et al. [26] in shallow coastal areas of the Baltic Sea (Fig. 1A-C). Each sampling station consisted of three subsamples taken within the *Z. marina* meadow (“inside”), approximately 15 m from the meadow (“edge”) and at least 100 m from the *Z. marina* meadow (“outside”) (Fig. 1D). Samples for DNA extraction from water and sediment were collected from each substation. For water, 100 mL syringes were used to sample ca. 5 cm from the *Z. marina* and 20 cm above the sediment surface. Sediment samples were collected using sterile plastic tubes by scraping the upper 1 cm at each station.

The *Z. marina* densities were counted in triplicate 20 × 20 cm squares. Leaf length was measured for 30 plants in each meadow. All samples were immediately transferred to a 4 °C cooler and stored (maximally 8 h) until processing. Salinity, temperature, and depth were measured using a CTD48M (Sea & Sun Technology). The dissolved oxygen and pH were measured using a HQ40D portables 2-channel multimeter. Phosphate (PO_4_^3−^), nitrate (NO_3_^−^), nitrite (NO_2_^−^), ammonium (NH_4_^+^), and silicate (SiO_2_) concentrations were measured using a Seal Analytical QuAAtro automated continuous flow analyzer (SEAL Analytical Ltd. Nordestedt. Germany). Chlorophyll-*a* (Chl-*a*) was measured fluorometrically using a 10- AU-005-CE fluorometer (Turner San Jose, USA).

To determine bacterial and eukaryotic community composition, water was filtered through 0.2 μm polyvinylidene fluoride membrane filters (Merck, Darmstadt, Germany) and shock frozen in liquid nitrogen. DNA was extracted after bead beating using a DNeasy PowerSoil Pro Kit Pro (Qiagen. Hilden. Germany) following the manufacturer’s protocol after grinding the filters. For the sediment samples, a subsample (300 mg) of the sediment was transferred into bead-beating tubes. The samples were placed on ice, sonicated twice for 7 min, and then subjected to bead-beating for 30 s at 4 m/s on a vortex adaptor. Subsequently, the manufacturer’s instructions were followed, and the DNA yield was quantified using the PicoGreen assay (Thermo Fisher, Waltham. USA).

The 16S rRNA genes were amplified using V3–V4 primers [29], and the 18S rRNA genes were amplified by targeting the eukaryotic V4 region [42]. The samples were sequenced on a MiSeq (Illumina Inc. San Diego. CA. US) for the 16S and 18S rRNA genes by SciLifeLab/NGI (Solna. Sweden). Amplicon sequencing data were processed using the workflow described by Riedinger et al. [26] (https://github.com/biodiversitydata-se/amplicon-multi-cutadapt). ASVs with similar taxonomic assignment were aggregated.

For the bacterial community, sequences assigned to Archaea, chloroplasts, and mitochondria were removed before analysis because the primers used covered only minor parts of these groups. After the removal of these groups, a total of 11,599,548 reads were available for the bacterial community (Appendix Fig. 1A). For the eukaryotic data, reads assigned to unclassified Opisthokonta, Fungi, Embryophyceae (including *Zostera* spp.), and Metazoa were excluded, resulting in a total of 20,702,520 reads (Appendix Fig. 1B).

### Statistical analysis

The amplicon sequence variant (ASV) richness (S_OBS_) and Shannon index were estimated using Explicet [43], which performs rarefaction-based analysis via bootstrapping. For all stations, bootstrap resampling was conducted at the size of the smallest library at the rarefaction point (Bacteria: 10,257 excluding two samples; Eukarya 25,417 excluding two samples) to compare ASVs between libraries with equal sampling efforts. The Kruskal–Wallis test and a *post hoc* Tukey`s pairwise test was used to determine significant differences between the numbers of ASVs in the samples. A sequential Bonferroni correction was used for multiple comparisons. For the sample groups, *p* > 0.01 indicated that the variances were not significantly different from each other.

Due to the large spread between individual sampling sequencing depth (Bacteria: 8,720 − 138,054 reads, Eukarya: 9,699 − 213,379 reads), quality-trimmed sequencing reads were transformed using a centered log-ratio (CLR) transformation. This compositional data analysis approach [44] separates data variance according to differences rather than abundances. PCA on the pairwise Aitchison distances (Euclidean distance on CLR-transformed data [45]) was used as an exploratory analysis of the microbiota composition [46]. Then, the data were split into sub-datasets, and each micro-environment was tested separately to assess the impact of environmental factors. The effects of environmental variables on the community composition patterns of water and sediment were analyzed using PERMANOVA and two-way PERMANOVA based on Aitchison distances. The number of permutations was set to 9999, and *p* < 0.01 was considered significant.

For analyzing differences between microbiota at different water depths, the averages of *Z. marina* abundance and length at the different sampling stations were calculated, with values above the average categorized as high and below the average as low (Appendix Table 1).

**Table 1:** Physicochemical parameters measured at the stations (see Fig. 1A-C). The substations “inside, edge, and outside” (see Fig. 1D) were averaged because the differences were minimal. *Zostera marina* shoot length was determined from inside samples. (Chl a = Chlorophyll a, DOC = dissolved organic carbon, PON = particular organic matter, DN = dissolved nitrogen, NA = not available)

	Sample	Salinity	Chl a (mg m^− 3^)	Depth^1^ (m)	Wave (m)	Wind (Bft)	PO_4_ (µmol l^− 1^)	Length^2^ (cm)	Temp (°C)	POC (µmol l^− 1^)	DOC (µmol l^− 1^)	NH_4_ (µmol l^− 1^)	PON (µmol l^− 1^)	DN (µmol l^− 1^)	SiO_2_ (µmol l^− 1^)
β – meso- haline	BV-21	6.7	1.6	3.3	0.3	3	0.5	39	16.4	21.4	378.1	1.3	3.9	18.4	15.8
	BV-18	6.1	2.3	2.6	0	0	NA	22	18.8	36.2	385.8	NA	5.2	18.3	9.0
	BV-16	6.7	1.5	3.2	0.1	1.8	0.2	42	17.8	21.6	354.8	1.2	3.6	19.7	10.0
	BV-15	6.8	2.4	2.4	0.2	2	0.1	28	19.0	31.5	372.8	0.6	4.6	19.3	9.8
	BV-14	6.3	3.1	4.5	NA	NA	0.1	38	18.7	61.4	426.1	NA	6.6	21.1	9.4
	BV-12	7.0	NA	2.0	0	0.3	NA	44	NA	NA	NA	NA	NA	NA	NA
	BV-11	7.6	19.7	2.2	0.5	4	0.5	53	20.0	265.3	614.8	0.8	34.9	28.0	43.3
	BV-9	7.7	2.3	2.9	0.9	4	0.3	44	20.4	37.5	373.9	NA	5.3	18.7	13.7
**Average**		**6.9**	**4.4**	**2.5**	**0.1**	**2.3**	**0.3**	**39**	**18.9**	**61.9**	**407.6**	**1.0**	**8.4**	**20.3**	**15.2**
α- meso- haline	BV-24	9.6	1.3	2.0	0.1	2	0.3	38	21.0	22.4	353.0	NA	3.7	18.3	9.5
	BV-22	11.7	NA	1.2	0.1	2.7	NA	74	NA	NA	NA	NA	NA	NA	NA
	BV-10	9.3	2.9	4.1	0.3	3	0.2	109	21.2	28.2	371.5	2.1	3.8	17.3	5.1
	BV-8	9.5	1.4	2.9	0.1	1	0.1	75	21.3	16.2	361.4	0.7	2.7	18.0	5.3
	BV-7	9.8	2.1	2.5	0.1	3	0.4	48	21.4	23.3	356.7	0.8	3.7	19.1	9.8
	BV-6	10.5	1.9	3.7	NA	NA	0.1	85	19.4	59.9	334.5	0.9	6.4	17.1	4.3
	BV-5	15.4	0.7	1.2	NA	NA	0.1	60	18.2	17.4	323.7	1.0	2.8	15.6	2.4
**Average**		**10.9**	**1.6**	**2.5**	**0.1**	**2.3**	**0.2**	**70**	**20.0**	**26.7**	**344.7**	**1.3**	**3.7**	**17.3**	**5.9**
**Total Average**		**8.7**	**3.1**	**2.7**	**0.2**	**2.1**	**0.3**	**53**	**19.4**	**45.4**	**378.2**	**1.2**	**6.2**	**18.9**	**10.9**

^1^ water depth; ^2^*Zostera marina* shoot length

## Results

### Characterization of sampling sites

The samples were categorized following a modified Venice system [47], which takes the salty divide into account (Table 1): alpha-mesohaline conditions (salinity 9.3–15.5; stations BV5, BV6, BV7, BV8, BV10, BV22, BV24) and beta-mesohaline conditions (salinity 6.1–7.7; stations BV9, BV11, BV12, BV14, BV15, BV16, BV18, BV21). Both alpha- and beta-mesohaline sites were characterized by an average depth of ~ 2.6 m, low waves (~ 0.2 m), and wind speed (2.1 Bft). The average temperature and salinity were higher at alpha-mesohaline conditions compared to beta-mesohaline conditions. Chl-*a*, POC, PON, and SiO2 were, on average, more than twice as high under beta-mesohaline conditions than under alpha-mesohaline conditions. In particular, the beta-mesohaline station BV11 (Greifswalder Bodden) where higher compared to the average chemical measurements for Chl-*a* (19.7 mg m^− 3^), PON (34.9 µmol L^− 1^), POC (265.3 µg L^− 1^), DOC (614.8 µmol L^− 1^) and SiO_2_ (43.3 µmol L^− 1^) indicating the presence of a phytoplankton bloom.

### Microbial patterns in sediment and water within and outside the *Zostera marina* meadows

**Fig. 2:**
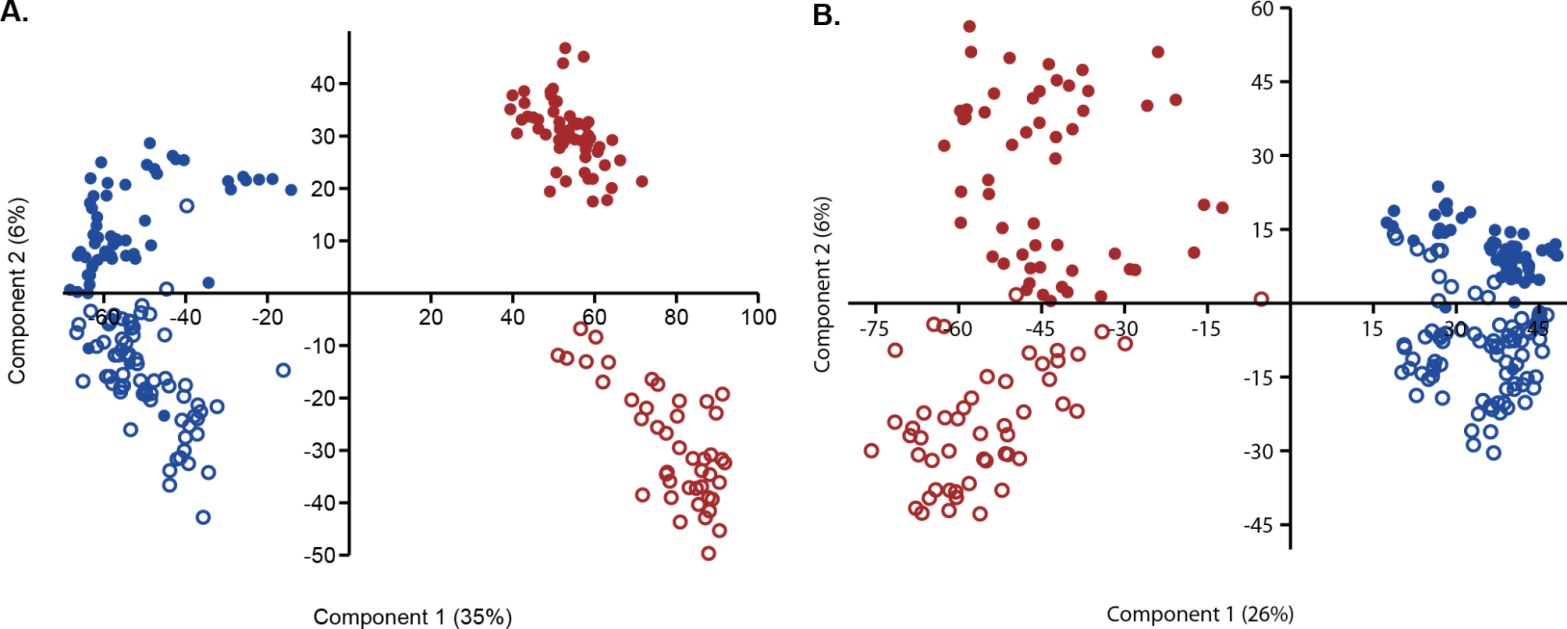
Principal component analysis of the (**A**) Bacterial community and (**B**) Eukaryotic community based on Aitchison distances in sediment (brown) and water (blue). Dots indicate samples taken under alpha-mesohaline conditions (salinity 9.3–16), and circles samples taken under beta-mesohaline conditions (salinity 6-7.8)

PCA of the bacterial (Fig. 2A) and eukaryotic (Fig. 2B) community composition showed a clear separation between the water and sediment microbiota along the first component (two-way PERMANOVA: Bacteria F = 131, *p* < 0.01; Eukarya F = 87, *p* < 0.01), independent of all other factors measured. Along the second component, a separation between alpha- and beta-mesohaline samples was apparent (two-way PERMANOVA: Bacteria F = 19, *p* < 0.01; Eukarya F = 16, *p* < 0.01). The interaction between the sediment/water and salinity category was also significant (two-way PERMANOVA: Bacteria F = 15, *p* < 0.01; Eukarya F = 11, *p* < 0.01). Because the sediment and water microbiota were clearly separated, we analyzed them separately. Envfit analysis of the sediment and water microbiota showed that the community composition was strongest associated (highest r²) with salinity (Table 2).

**Table 2:** Results of the envfit analysis. PC1 and PC2 show the relationships of the environmental variables with the first and second PCA axes (based Aitchison distance). R² is the variation explained by the multiple regression model. Pr(> r) indicates the significance of the multiple regression. Significance codes: 0 ‘***’ 0.001 ‘**’ 0.01 ‘*’ 0.05. (A) bacterial community water samples and (B) bacterial community sediment samples (C) eukaryotic community water samples and (D) eukaryotic community sediment samples

	PC1	PC2	*r* ^2^	Pr(> *r*)
(**A**)				
Temperature	-0.54	-0.84	0.07	0.016*
Salinity	-0.99	0.05	0.69	0.001***
PON	0.94	0.35	0.18	0.001***
Chl- *a*	0.99	0.17	0.17	0.001***
POC	0.83	0.56	0.26	0.001***
(**B**)				
Temperature	0.98	-0.19	0.24	0.001***
Salinity	-0.99	0.01	0.67	0.001***
PON	-0.52	-0.86	0.09	0.005**
Chl- *a*	-0.51	-0.86	0.10	0.001***
POC	-0.47	-0.88	0.08	0.005**
(**C**)				
Temperature	-0.36	-0.93	0.15	0.001***
Salinity	-0.99	-0.10	0.54	0.001***
PON	0.64	-0.76	0.46	0.001***
Chl-*a*	0.64	-0.77	0.46	0.001***
POC	0.65	-0.75	0.45	0.001***
(**D**)				
Temperature	-0.83	0.56	0.26	0.001***
Salinity	-0.95	0.30	0.73	0.001***
PON	0.20	-0.98	0.06	0.041*
Chl- *a*	-0.19	-0.98	0.06	0.058
POC	-0.17	-0.98	0.06	0.049**

**Fig. 3:**
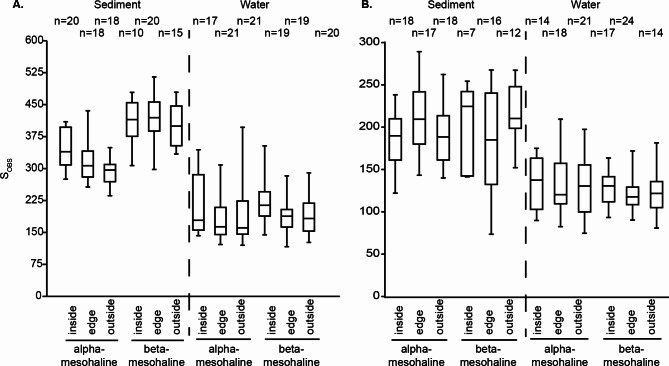
Alpha diversity (S_obs_) of the (**A**) bacterial community and (**B**) eukaryotic community

To examine the potential influence of the presence of *Z. marina* in water and sediment microbiota, samples were divided according to proximity to *Z. marina* meadows (inside, edge, and outside) and analyzed separately for alpha- and beta-mesohaline conditions and for water and sediment samples. PC analysis and PERMANOVA revealed no significant effect of proximity to *Z. marina* on the water bacterial and eukaryotic community composition under neither alpha (F = 0.9, *p* = 0.54) nor beta (F = 1.1, *p* = 0.23)-mesohaline conditions (Appendix Fig. 2C, D, G, H). The interaction effect between salinity and proximity to *Z. marina* was also nonsignificant (two-way PERMANOVA: F = 0.59, *p* = 0.72). Similarly, no significant differences in the water column alpha-diversity (neither S_OBS_ nor Shannon index) in relation to *Z. marina* proximity were detected under either alpha-mesohaline or beta-mesohaline conditions (Kruskal-Wallis test: *p* > 0.01; Fig. 3, Appendix 3).

**Fig. 4:**
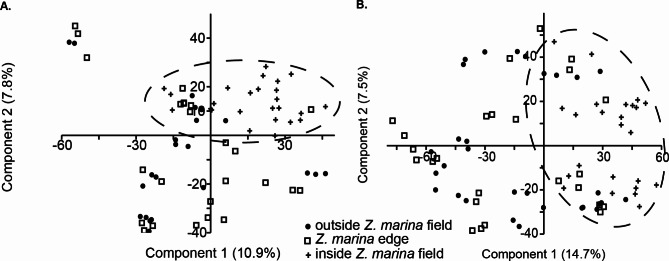
Effect of *Zostera marina* at alpha-mesohaline conditions on the sedimental (**A**) bacterial community and (**B**) sediment eukaryotic community

In contrast, both the bacterial and eukaryotic community compositions of the sediment samples were significantly different inside the *Z. marina* meadow compared to on the edge or outside the meadows for the alpha-mesohaline samples (F = 2.7, *p* < 0.01; Fig. 4, Appendix 2 B, F). However, in the lower saline beta-mesohaline samples, the effect of *Z. marina* proximity was not observed on the microbiota (F = 1.0 *p* = 0.3; Appendix 2 A, E). The interaction effect between salinity and proximity to *Z. marina* was insignificant (two-way PERMANOVA: F = 0.84, *p* = 0.08). Similar to the effects on the community composition, the presence of *Z. marina* affected the alpha-diversity (S_OBS_ and Shannon index) of the bacterial sediment community, only at alpha-mesohaline conditions, where a significantly higher S_OBS_ was observed inside the *Z. marina* meadow than at the edge and outside (Kruskal-Wallis test: *p* < 0.01; Fig. 3). Interestingly, no significant differences were found in the S_OBS_ and Shannon index between the inside-edge-outside samples of the eukaryotes for sediment or for the water samples (Kruskal-Wallis test: *p* > 0.01, Fig. 3, Appendix 3).

*Z. marina* leaf length differed significantly between alpha and beta-mesohaline conditions (*p* < 0.01, Appendix 4). Additionally, there was a significant difference in the bacterial and eukaryotic communities among the different *Z. marina* length categories (Table 3). In addition, *Z. marina* abundance had a significant influence (*p* < 0.01) on the microbiota in contrast to water depth.

**Table 3:** PERMANOVA of the bacterial (A) and eukaryotic (B) community compositions at water depth and of *Z. marina* abundance and leaf length categories. (A)

	water depth		*Z. marina* abundance		*Z. marina* leaf length	
	F-value	*p*-value	F-value	*p*-value	F-value	*p*-value
(**A**)						
sediment	1.73	0.039	3.33	> 0.001	3.35	> 0.001
water	1.60	0.018	2.61	> 0.001	2.31	> 0.001
(**B**)						
sediment	1.43	0.060	2.54	> 0.001	2.98	> 0.001
water	1.48	0.043	2.54	> 0.001	3.34	> 0.001

### Differences in taxonomic composition

**Fig. 5:**
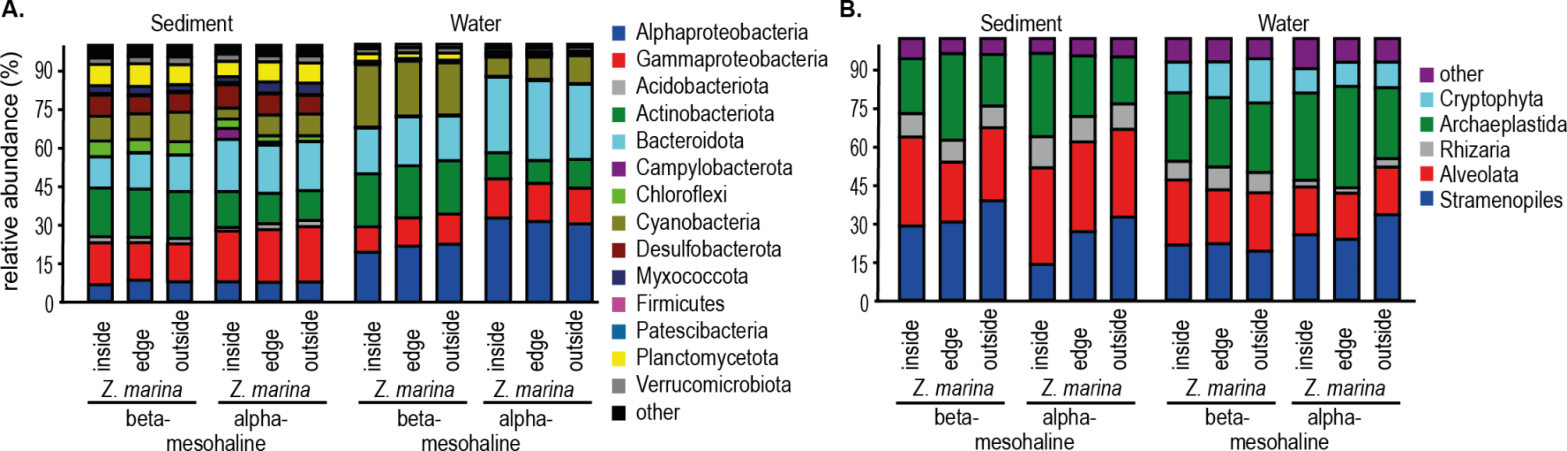
Bacterial phyla/classes (**A**) and eukaryotic phyla/classes (**B**) with abundances > 1% in the sediment and water at alpha-mesohaline and beta-mesohaline conditions

The most abundant (> 1%) phyla/classes of the bacterial communities in our study included Bacteroidetes (12–31%), Alphaproteobacteria (7–33%), and Gammaproteobacteria (10–22%; Fig. 5). The sediment samples contained higher relative abundances of Gammaproteobacteria (sediment 18% vs. 13% water), Planctomycetota (sediment 8% vs. 2% water), and Desulfobacterota (sediment 8% vs. 1% water). Alphaprotoeobacteria (sediment 7% vs. 27% water), Bacteroideta (sediment 17% vs. 24% water), Myxococcota (sediment 5% vs. > 1% water), Chloroflexi (sediment 4% vs. > 1% water), and Cyanobacteria (sediment 9% vs. 16% water) differed. Gammaproteobacteria (beta-mesohaline 15% vs. 21% alpha-mesohaline), Actinobacteria (beta-mesohaline 19% vs. 12% alpha-mesohaline) and Bacteroideta (beta-mesohaline 14% vs. 20% alpha-mesohaline) differed between the alpha and beta-mesohaline conditions. Within the *Z. marina* meadow under alpha-mesohaline conditions, a high abundance of Campylobacterota (4% inside vs. > 1% outside) was observed. The alpha-mesohaline and beta-mesohaline water bacterial community compositions also differed. Alphaproteobacteria (beta-mesohaline 21% vs. 32% alpha-mesohaline) and Bacteroidota (beta-mesohaline 18% vs. 30% alpha-mesohaline) were more abundant under alpha-mesohaline conditions, whereas Actinobacteria (beta-mesohaline 21% vs. 10% alpha-mesohaline) and Cyanobacteria (beta-mesohaline 22% vs. 9% alpha-mesohaline) were more abundant under beta-mesohaline conditions.

**Fig. 6:**
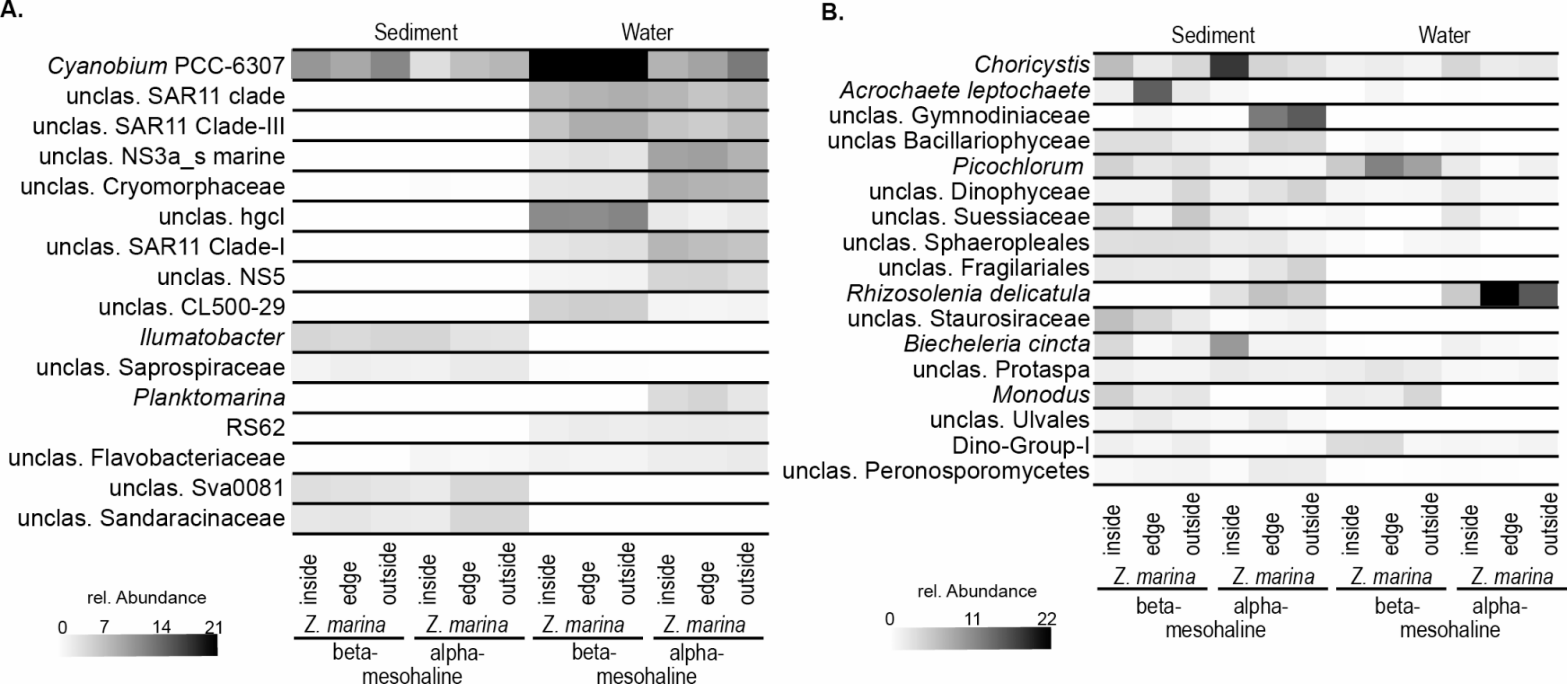
Heatmap of the relative abundances of (**A**) bacterial and (**B**) eukaryotic amplicon sequence variants (ASVs) > 1% in sediment and water at alpha-mesohaline and beta-mesohaline conditions. (unclas. = unclassified)

On a finer taxonomic level, *Cyanobium* was most abundant ASV in the water column (max = 21%) especially under beta-mesohaline conditions, but it was also abundant in all other habitats (Fig. 6A). The abundant bacterial groups typically found in the water column were SAR11 clades, NS3a, NS5, SAR86, and *Planktomarina* under alpha-mesohaline conditions, whereas hgcI (beta-mesohaline 9.3% vs. 2.1% alpha-mesohaline) and CL500-29 (beta-mesohaline 4.8% vs. 1.1% alpha-mesohaline) were more abundant under beta-mesohaline conditions. The bacterial communities in the sediments were dominated by *Cyanobium*, *Illumatobacter*, unclassified Saprospiraceae, and the desulfosarcinal Sva0081 group. The bacterial genus Campylobacterota with higher abundances inside the *Z. marina* meadow at alpha-mesohaline conditions was mostly assigned to *Sulfurovorum*. Despite having a low overall abundance (0.8%), the genus has an abundance of 4% within the meadow. Representatives of the genus *Sulfurovorum* are able to oxidize sulfur.

Similar to the bacterial community composition, the community of microbial eukaryotes differed among the sediment and water samples (Fig. 6B). In water, *Picochlorum* and *Monodus* were abundant genera at beta-mesohaline conditions. *Rhizosolenia delicatula* was abundant under alpha-mesohaline conditions in water and sediment (beta-mesohaline 0% vs. 4.7% and 13.2% alpha-mesohaline). *Choricystis* sp. were more abundant inside (inside 13.3% vs. 3.6% outside) the *Z. marina* meadow and Gymnodiniaceae, rather than outside the meadow (inside 0.3% vs. 12.0% outside).

## Discussion

*Z. marina* meadows stabilize sediments, provide food and shelter, and recycle nutrients, providing critical aquatic ecosystem services. *Z. marina* has been suggested to impact its environment beyond the direct *Z. marina* leaves (phyllosphere) [14–17, 19] and roots (rhizosphere) [11–13]. Our results showed a limited effect of *Z. marina* on the surrounding water and sediment microbiota since only at alpha-mesohaline conditions in sediments a significant effect of *Z. marina* on the microbiota was observed. Therefore, the first hypothesis was partially supported. Previous studies also observed an influence of *Z. marina* under saltwater conditions [12–14, 16, 17, 48–50].

The missing effect of *Z. marina* meadows at lower salinity could be related to the symbiont (microbiota) or host (*Z. marina)*. Among the mechanisms by which *Z. marina* influences the surrounding microbiome is the release of bioavailable carbon and the resulting accumulation of microbial grazers [51] and bacteria that excrete antimicrobial substances [52]. However, these effects appear to be local and may be detected only when the phyllosphere or the rhizosphere is directly sampled. The release of oxygen from the roots of *Z. marina* has also been shown to influence the surrounding microbiota [6]. However, since we sampled oxygenated top-layer sediments, the impact of released oxygen was likely minor. Another proposed mechanism is the absorption of pathogens by biofilms [53], which was not observed in in lower salinities [26].

Mechanisms of *Z. marina* influencing the surrounding sediment and water include a reduction of wave energy and increase sedimentation as a physical effect of *Z. marina* canopy structure (“canopy effect”) [54, 55]. Under beta-mesohaline conditions, *Z. marina* has a lower productivity that is connected with a significant reduced leaf length (Table 1, Appendix 4). Shorter leaves also reduce the canopy effect, which reduces the capacity of *Z. marina* to trap fine sediments, including particle-attached bacterial communities [56] from the water column. At higher salinities, longer leaves are more likely to trap particle-attached bacterial communities, resulting in a significant change in the meadow surface sediment bacterial community [18]. Accordingly, a correlation between the canopy structure of *Z. marina* and the microbiota was previously reported [5, 18, 55, 57]. However, other local factors, in addition to salinity, including sediment type, history, and wave exposure, can also influence leaf length [58, 59].

In contrast to previous studies, no specific bacterial community was found on the edge of a *Z. marina* meadow in the water and sediment [5, 18]. Therefore, the second hypothesis was rejected. This could be attributed to the fact that the edges of the *Z. marina* meadow in our study were predominantly patchy and sampled in a distance of 15 m from the main meadow. Samples closer to the meadow may show a different result. We also found higher sediment bacterial diversity within the *Z. marina* meadows than outside under alpha-mesohaline conditions. This increased diversity of microbiota in close proximity to plant roots due to exudates was previously described as the “rhizosphere effect” [60]. Other studies [5, 61, 62] in contrast suggested higher diversity in bulk sediment outside the *Z. marina* meadow because the rhizosphere selects microorganisms from the bare sediment and reduces overall microbial diversity. The discrepancy may be connected to the brackish conditions in our study compared with the saltwater conditions in other studies and the fact that we did not sample the rhizosphere, but rather surface sediment in the *Z. marina* meadow.

The missing “*Z. marina* meadow”-effect of the water bacterial and microeukaryotic communities is in line with studies on the San Diego coastal water, in which the location of the sampling site was identified as the major driver of the bacterial community composition rather than proximity to the *Z. marina* meadow [15]. Other studies have reported a reduction in colony-forming units in *Z. marina* beds compared with surrounding water [20–23]. However, these studies were conducted at higher salinity. The reduced canopy effect in our study may also have reduced the effects on the water bacterial community. In addition to the canopy effect, a potential mechanism by which *Z. marina* directly influences water microbial communities is the release of exudates as dissolved organic matter (DOM) [6, 63]. The high DOM concentration in the Baltic Sea [64], which is caused by terrestrial influences, especially in the littoral zone, may mask the effects of DOM release on aquatic microbial communities. Moreover, DOM can be confounded by salinity; therefore, more studies, including experiments under controlled conditions, are needed to support the observed trends in this study.

### Other effects on the microbiota

The bacterial and eukaryotic communities in the sediment and water were significantly different (Fig. 2). This is in accordance with previous studies in which the effect of microenvironment type has been shown to have a stronger impact on determining the bacterial community composition than salinity [14, 17, 65–67]. The second most important factor influencing microbiota structure was salinity. Based on the salinity range covered (salinity 6–15), clustering of the samples according to alpha-mesohaline and beta-mesohaline conditions was expected [28–30, 34, 68]. The S_OBS_, which reflects microbial richness, was highest in the sediment (Fig. 3), which is consistent with previous studies [5, 29, 30]. In contrast to the species minimum at the salty divide (“horohalinicum”) observed for macroorganisms [34] and phytoplankton [68], the bacterial and microeukaryotic community species richness in the water was rather constant at different salinities (Fig. 3). No significant effects on bacterial and microeukaryotic S_OBS_ have been reported in previous studies [29, 31, 69, 70]. In accordance with these studies, a shift was observed between the dominance of clades present in saltwater, including SAR11 clade I, NS3a, NS5, SAR86, and *Planktomarina*, and the dominance of clades typically found in freshwater, including HGC-I, SAR11-III, and CL500-29, at the salty divide. Most ASVs were assigned only to their taxonomic genus due to the short-read length of the method and the lack of species descriptions in current databases [71]. This resulted in a relatively low diversity of taxonomic genera and many undescribed taxonomic clades (Fig. 6).

For the water microbiota, a rather gradual change in community composition was observed with changing salinity, whereas the distinction between the alpha- and beta-mesohaline microbial communities in sediments was more pronounced (Fig. 2). This could be connected with a higher resistance of sediment bacterial communities to changes in salinity [66] and stronger mixing of water masses than in sediment, but also with differences in the sediment characteristics of the two salinity conditions.

## Conclusion

A global effort is underway to restore coastal and estuarine ecosystems, and a major part of this effort is the reestablishment of *Z. marina* [72]. Our findings provide fundamental information about the impact of *Z. marina* on sediment and water microbiota under brackish conditions. We found that the influence of *Z. marina* on the microbiota was lower under brackish conditions, especially below a salinity of 9. The shift in salinity has a significant effect on both the microbiota and host appearance. Because leaf length can affect the impact on the sediment bacterial community in a *Z. marina* meadow, we suggest that the canopy effect is pivotal to the influence of *Z. marina* meadows on the surrounding environment. This finding supports the previous hypothesis that the *effects of Z. marina* are rather passive [18, 55]. The results should be considered when managing brackish *Z. marina* meadow ecosystem services in brackish environments, such as the Baltic Sea, Black Sea, and larger estuaries.

## Electronic supplementary material

Below is the link to the electronic supplementary material.

Supplementary Material 1

## Data Availability

The datasets supporting the conclusions of this article are available in the European Nucleotide Archive under accession number PRJEB68222, which is in compliance with the Minimum Information about any (X) Sequence (MIxS) standard through the brokerage service GFBio93. Environmental data are available from IOWMeta (doi.io-warnemuende.de/10.12754/data-2023-0010).
