# Supplementary material for: Low impact of Zostera marina meadows on sediment and water microbiota under brackish conditions
Source: Environ Microbiome. 2025 Jan 11;20:2. doi: 10.1186/s40793-024-00662-6 (PMC11724437; doi:10.1186/s40793-024-00662-6)
Supplement: Supplementary file 1 — Supplementary Material 1 [file 40793_2024_662_MOESM1_ESM.pdf]

Appendix

Low impact of Zostera marina meadows on sediment and water microbiota under brackish conditions

Herlemann et al.

Appendix table1. Growth characteristic, and water depth of *Zostera marina* on the sampling stations.

Row Labels	water depth (m)	category depth	Average of count *Zostera marina* 0,04 m²	category count	Average of abundance *Zostera marina* (ind m-1)	category abundance	Average of Length *Zostera marina* leaves	category lenght
BV-05-A	1.2	shallow	42.3	high	1058.3	high	60.0	long
BV-06-A	3.7	deep	18.3	high	458.3	high	85.4	long
BV-07-A	2.5	shallow	27.0	high	675.0	high	48.3	short
BV-08-A	2.9	deep	16.0	low	400.0	low	74.6	long
BV-09-A	2.9	deep	16.0	low	400.0	low	43.7	short
BV-10-A	4.1	deep	5.0	low	125.0	low	108.8	long
BV-11-A	2.2	shallow	6.5	low	162.5	low	52.9	short
BV-12-A	2.0	shallow	9.7	low	241.7	low	43.8	short
BV-14-A	4.5	deep	17.5	low	437.5	low	38.4	short
BV-15-A	2.4	shallow	14.7	low	366.7	low	28.2	short
BV-16-A	3.2	deep	24.5	high	612.5	high	41.8	short
BV-18-A	2.6	shallow	9.0	low	225.0	low	22.5	short
BV-21-A	3.3	deep	18.7	high	466.7	high	39.3	short
BV-22-A	1.2	shallow	27.0	high	675.0	high	74.3	long
BV-24-A	2.0	shallow	20.0	high	500.0	high	38.4	short
Average	2.7		18.1		453.6		53.3	

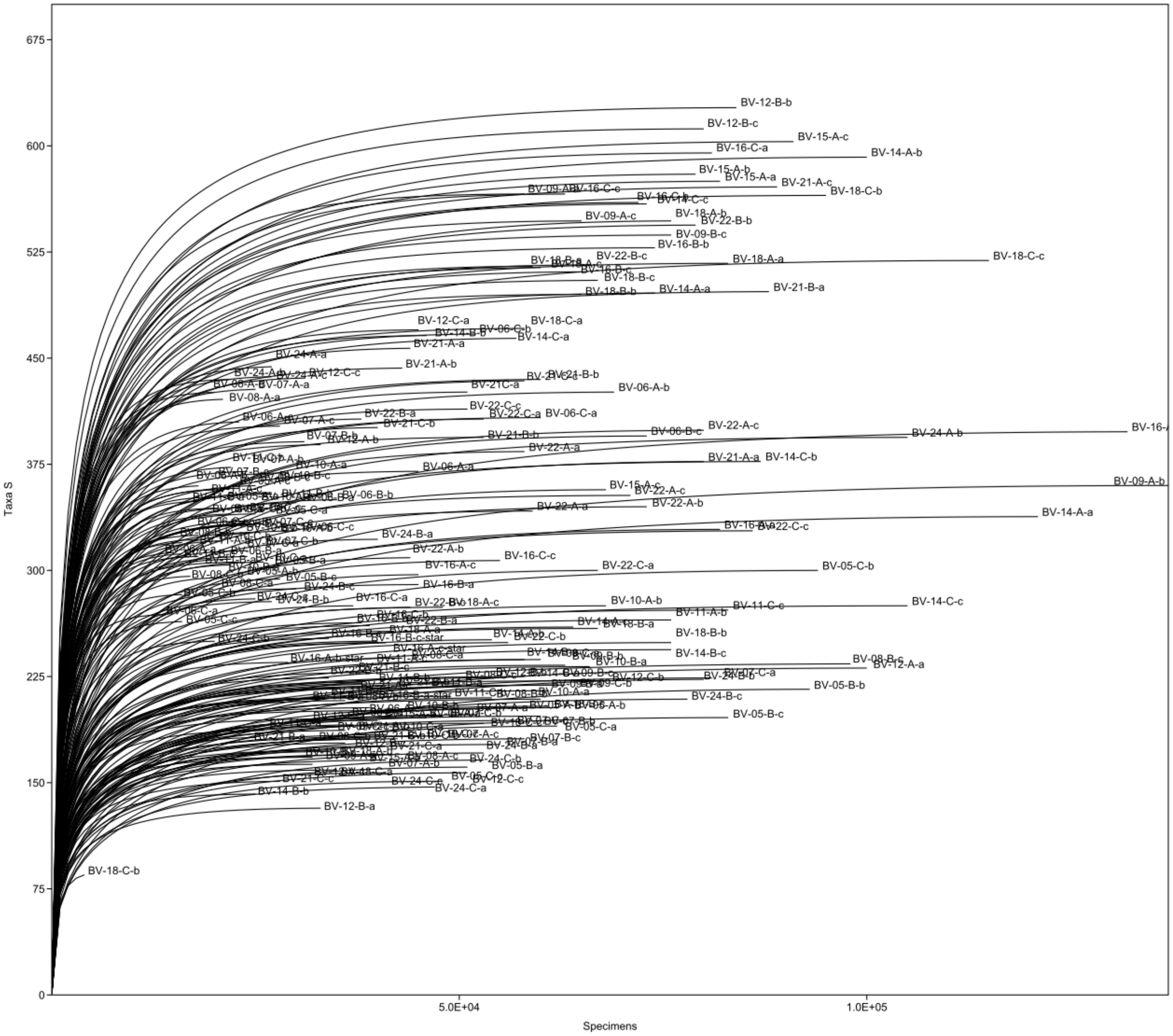

Appendix figure 1A. Rarefaction curves of the bacterial communities of individual samples

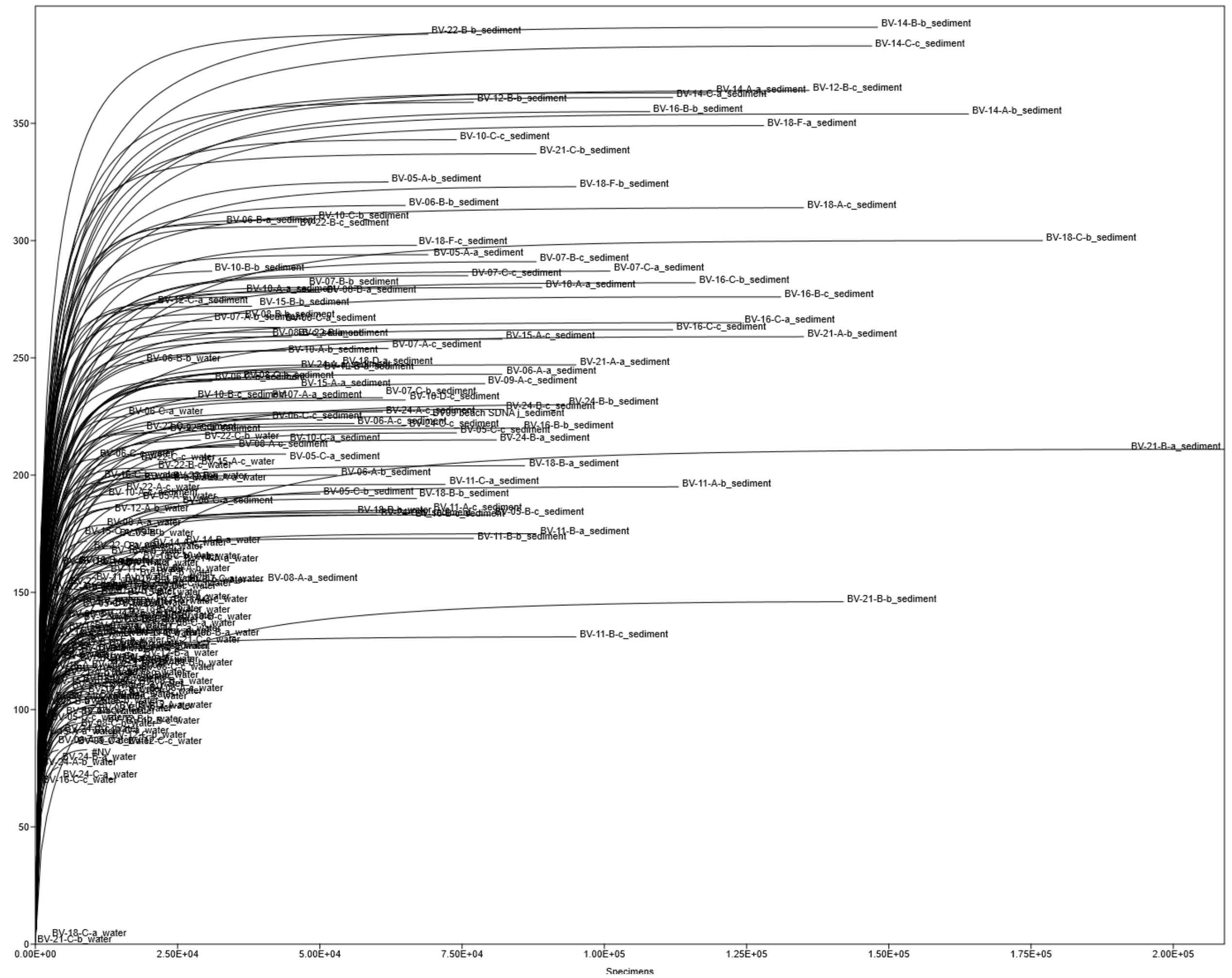

Appendix figure 1B. Rarefaction curves of the bacterial communities of individual samples

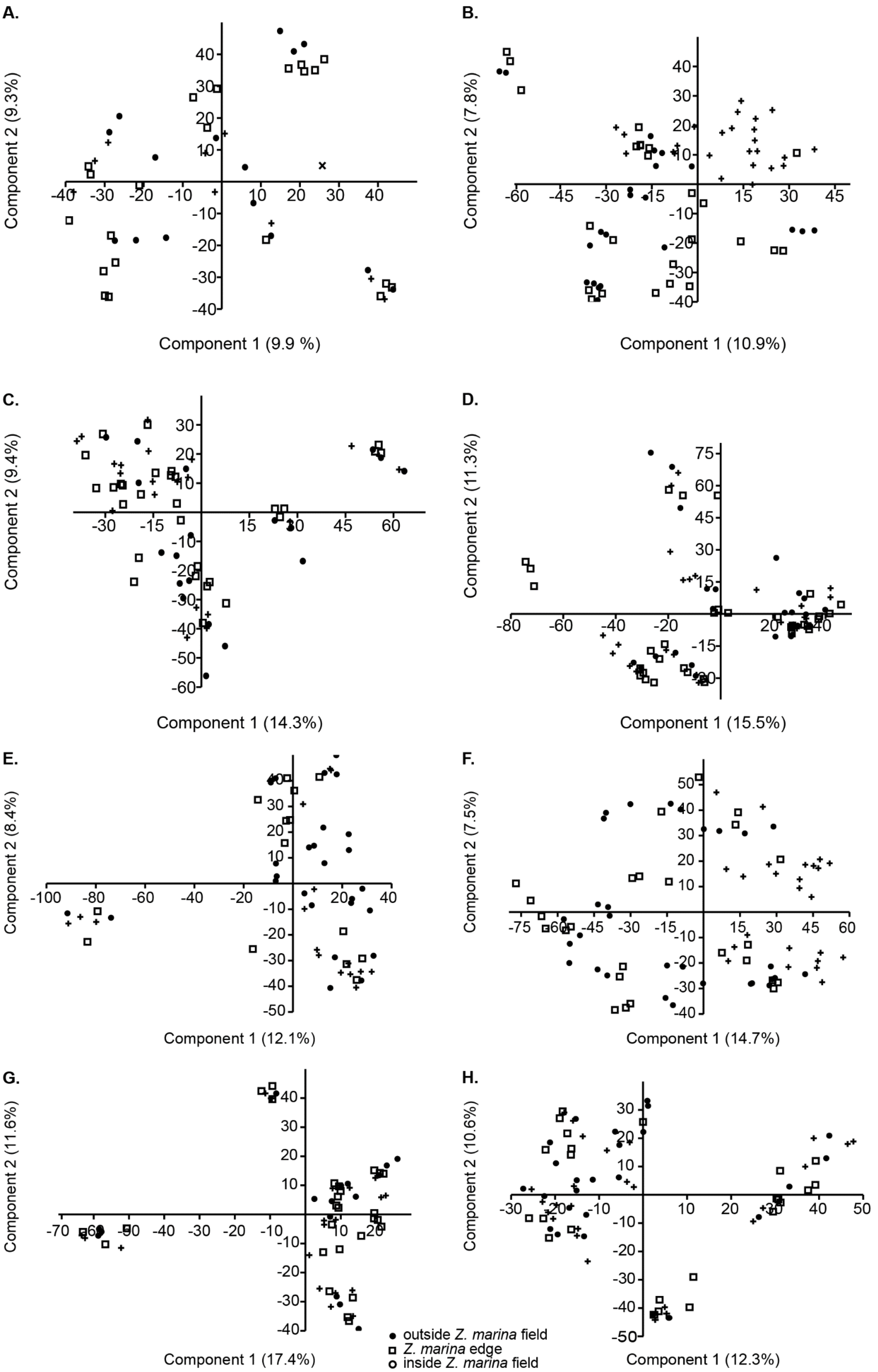

Appendix Figure 2. Influence of *Zostera marina* on the (A) bacterial communities in the sediment at beta-mesohaline (B) bacterial communities in the sediment at alpha-mesohaline (C) bacterial communities in the water at beta-mesohaline (D) bacterial communities in the water at alpha-mesohaline conditions (E) eukaryotic communities in the sediment at beta-mesohaline (F) eukaryotic communities in the sediment at alpha-mesohaline (G) eukaryotic communities in the water at beta-mesohaline (H) eukaryotic communities in the water at alpha-mesohaline conditions.

A. Bacterial Shannon index

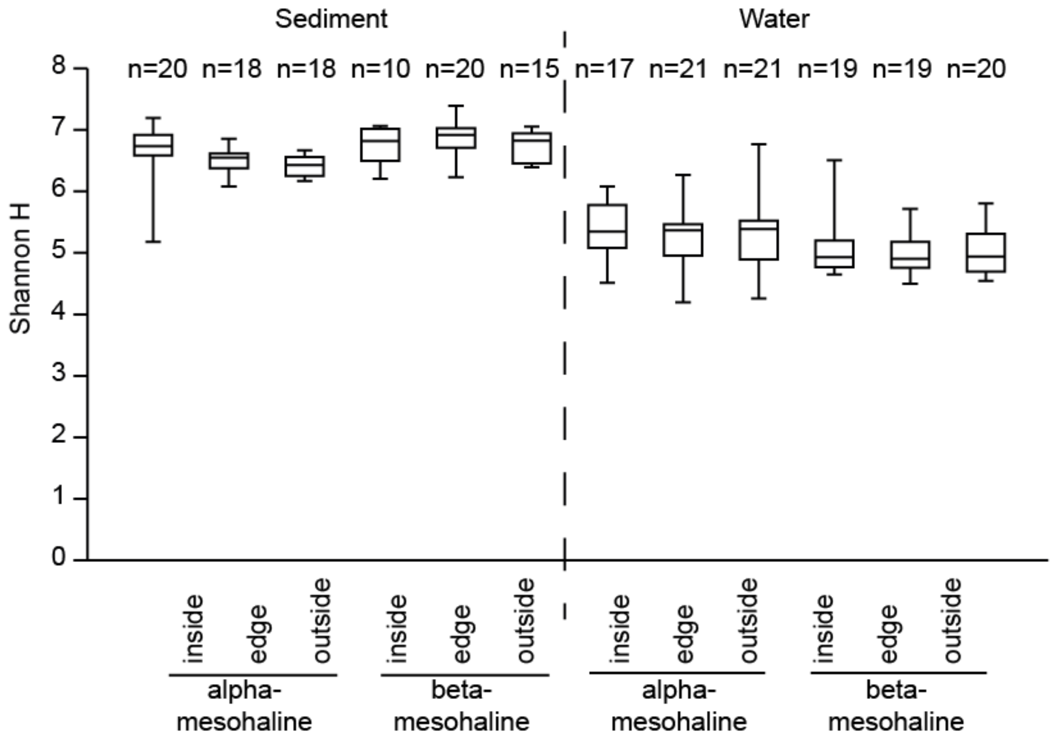

B. Microeukaryotal Shannon index

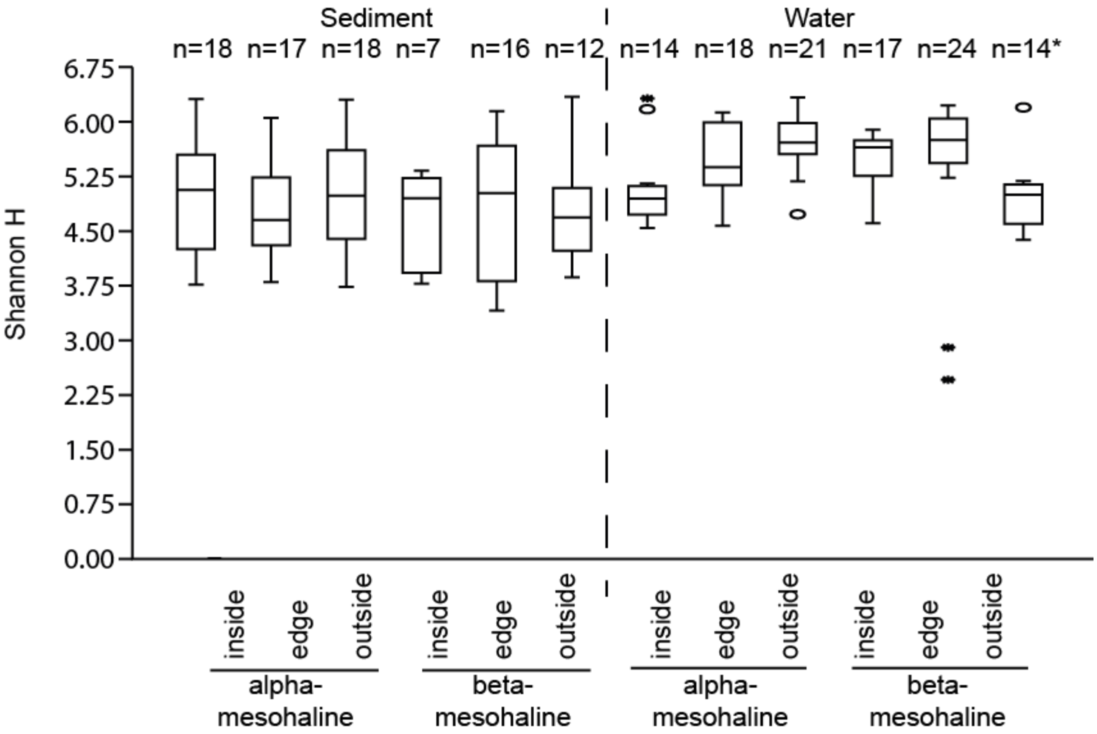

Appendix Figure 3.(A) Boxplot of the bacterial Shannon index. (B) Boxplot of the eukaryotic Shannon index

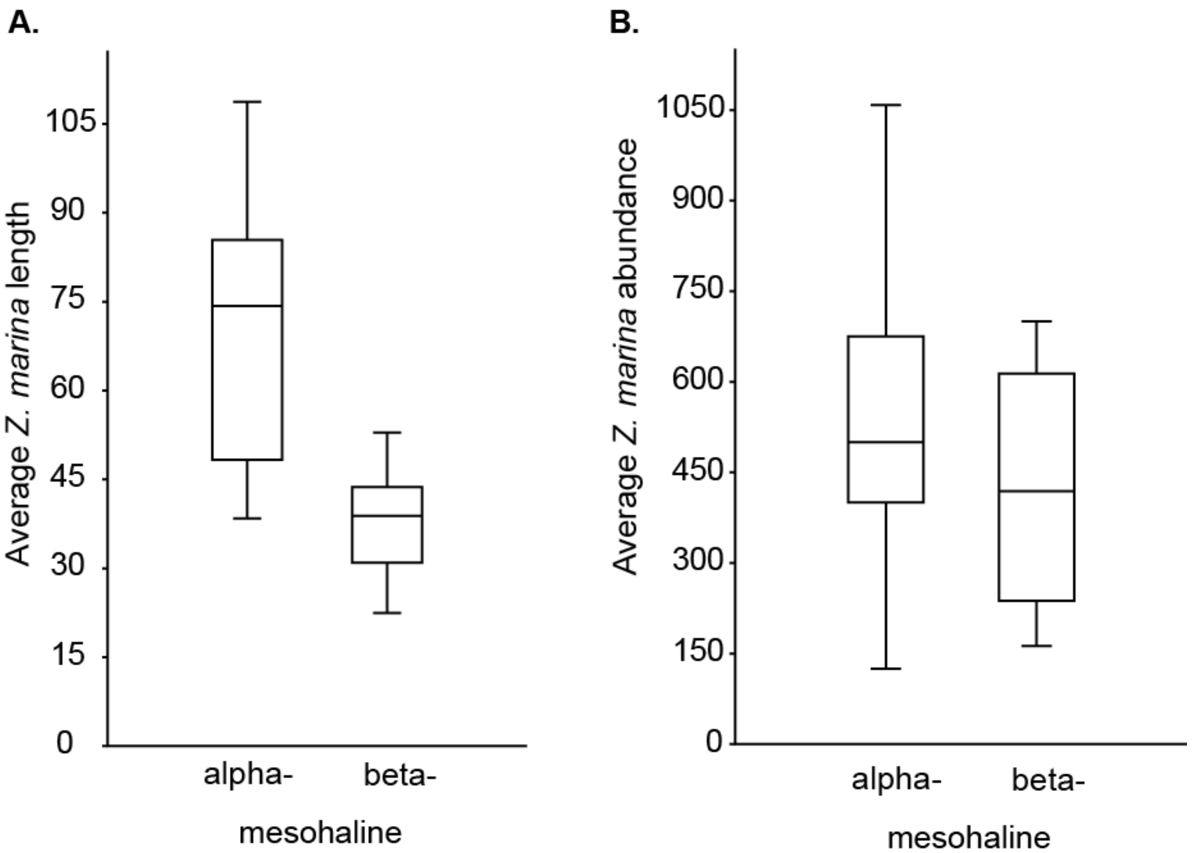

Appendix figure 4. (A) *Zostera marina* leaf lenght and (B) *Z. marina* abundance at alpha mesohaline and beta mesohaline conditions.
